# CFTR Is a Negative Regulator of NFκB Mediated Innate Immune Response

**DOI:** 10.1371/journal.pone.0004664

**Published:** 2009-02-27

**Authors:** Neeraj Vij, Steven Mazur, Pamela L. Zeitlin

**Affiliations:** Department of Pediatric Respiratory Sciences, The Johns Hopkins School of Medicine, Baltimore, Maryland, United States of America; LMU University of Munich, Germany

## Abstract

**Background:**

Dysfunctional CFTR in the airways is associated with elevated levels of NFκB mediated IL-8 signaling leading to neutrophil chemotaxis and chronic lung inflammation in cystic fibrosis. The mechanism(s) by which CFTR mediates inflammatory signaling is under debate.

**Methodology/Principal Findings:**

We tested the hypothesis that wt-CFTR down-regulates NFκB mediated IL-8 secretion. We transiently co-expressed wt-CFTR and IL-8 or NFκB promoters driving luciferase expression in HEK293 cells. Wt-CFTR expression in HEK293 cells suppresses both basal and IL1β induced IL-8, and NFκB promoter activities as compared to the control cells transfected with empty vector (p<0.05). We also confirmed these results using CFBE41o- cells and observed that cells stably transduced with wt-CFTR secrete significantly lower amounts of IL-8 chemokine as compared to non-transfected control cells. To test the hypothesis that CFTR must be localized to cell surface lipid rafts in polarized airway epithelial cells in order to mediate the inflammatory response, we treated CFBE41o- cells that had been stably transduced with wt-CFTR with methyl-β-cyclodextrin (CD). At baseline, CD significantly (p<0.05) induced IL-8 and NFκB reporter activities as compared to control cells suggesting a negative regulation of NFκB mediated IL-8 signaling by CFTR in cholesterol-rich lipid rafts. Untreated cells exposed to the CFTR channel blocker CFTR-172 inhibitor developed a similar increase in IL-8 and NFκB reporter activities suggesting that not only must CFTR be present on the cell surface but it must be functional. We verified these results *in vivo* by comparing survival, body weight and pro-inflammatory cytokine response to *P. aeruginosa* LPS in CFTR knock out (CFKO) mice as compared to wild type controls. There was a significant (p<0.05) decrease in survival and body weight, an elevation in IL-1β in whole lung extract (p<0.01), as well as a significant increase in phosphorylated IκB, an inducer of NFκB mediated signaling in the CFKO mice.

**Conclusions/Significance:**

Our data suggest that CFTR is a negative regulator of NFκB mediated innate immune response and its localization to lipid rafts is involved in control of inflammation.

## Introduction

Cystic fibrosis (CF) is an autosomal recessive disorder caused by mutations in the gene encoding the CF transmembrane conductance regulator (CFTR), a cAMP dependent and ATP-gated chloride channel that regulates epithelial surface fluid secretion in respiratory and gastrointestinal tracts [1]. Deletion of phenylalanine at position 508 (ΔF508) in CFTR is the most common cystic fibrosis causing mutation, resulting in a temperature sensitive folding defect, retention of the protein in the endoplasmic reticulum (ER) and subsequent degradation by the proteasome [2]. CF patients exhibit a typical phenotype that is characterized by persistent pulmonary infections, leading to pulmonary failure and death. Bronchoalveolar fluid (BAL) in CF patients contains increased levels of proinflammatory cytokines and neutrophils. CF cells have increased basal levels of pro-inflammatory C-X-C chemokine, interleukin (IL)-8, attributed to activated NFκB [3].

IL-8, the C-X-C chemokine, is a potent chemoattractant for neutrophils [4] that has been implicated in a number of inflammatory diseases, such as cystic fibrosis (CF) [5], adult respiratory distress syndrome [6], chronic obstructive pulmonary disease (COPD), and asthma [7]. The airway epithelium is one of the several sources of IL-8 in the airway [8]. The airway epithelium serves as a barrier against invading microorganisms. Airway epithelial release of IL-8 contributes to host defense by promoting neutrophil chemotaxis and airway inflammation. The exaggerated inflammatory responses in chronic diseases such as CF contribute to neutrophil-driven lung destruction [9]–[11]. Several cytokines such as IL1-β, tumor necrosis factor (TNF)-α, interferon (IFN)-γ and bacterial products, induce NFκB mediated IL-8 release from airway epithelial cells [12], [13], thus exacerbating the baseline inflammatory milieu in CF.

Although it has been almost two decades since the identification of the CFTR gene, we still do not fully understand the progression from chronic inflammation to bronchiectasis and end-stage CF lung disease[14], [15]. Excessive inflammation in the CF airway may be largely responsible for the development of bronchiectasis, but it has not been clear if chronic infection is always a factor[16], [17]. Several studies have shown that dysfunctional CFTR in the airways is associated with the elevated levels of NFκB mediated IL-8 signaling leading to neutrophil chemotaxis and chronic lung inflammation [14]. However, there is little consensus on the mechanism(s) which link mutant CFTR to chronic lung inflammation. In this study, we tested the hypothesis that functional CFTR on cell surface and its localization to cholesterol-rich lipid rafts is required for controlling both NFκB activity and downstream inflammatory signaling. Our results show that the expression of functional CFTR on cell surface negatively regulates the NFκB mediated innate immune response.

## Results

### CFTR negatively regulates NFκB and IL-8 signaling

**Figure 1:**
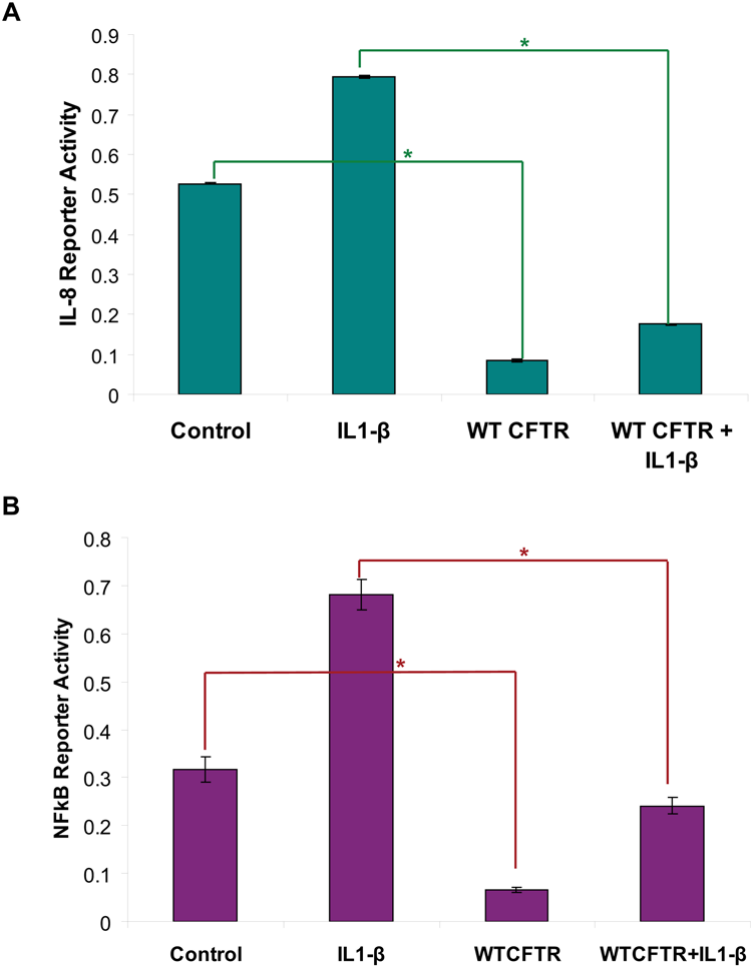
CFTR downregulates IL-8 and NFκB reporter activities. HEK293 cells were transiently transfected with wt-CFTR and IL-8 (A) or NFκB (B) reporter constructs together with a renila luciferase internal control plasmid (n = 3). The cells were induced with 1 ng/ml IL1-β overnight. Data are normalized to the renila luciferase internal control and expressed as mean±SD. A. IL-8 promoter driven luciferase expression. IL1-β stimulates IL-8 promoter activity and wt-CFTR blunts both control and IL1-1β driven IL-8 (*p<0.05). B. NFκB driven luciferase expression. IL-1β stimulates NFκB promoter activity and wt-CFTR blunts both control and IL-1-β driven NFκB (*p<0.05).

**Figure 2:**
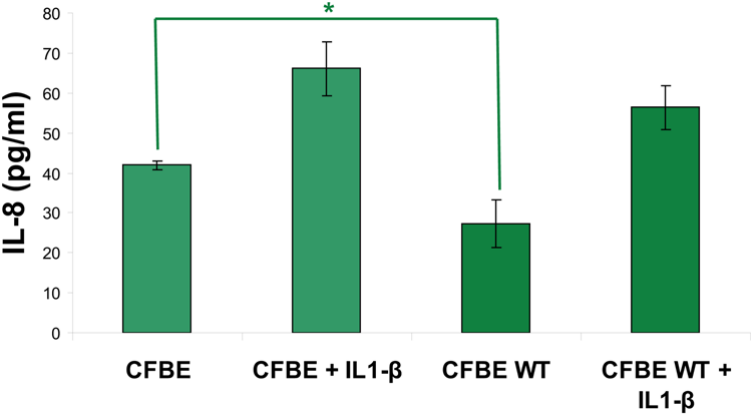
CFTR downregulates IL-8 chemokine secretion. CFBE41o- control and cells stably transduced with wt-CFTR were induced with 1 ng/ml IL1-β and IL-8 chemokine secretion in media was calculated after overnight treatment. Data are shown as mean±SD of three experiments. IL1-β stimulates IL-8 chemokine secretion while wt-CFTR blunts both control and IL1-1β driven IL-8 chemokine secretion (*p<0.05).

We and others have demonstrated that epithelial secretion of interleukin (IL)-8 is elevated at baseline in bronchial epithelial cells from human CF subjects [18]–[20]. This hyper-inflammatory response was also observed in both human CF respiratory epithelial cell lines[21] and in CF transmembrane conductance regulator deficient homozygous mice (CFTR−/−) compared to normal controls after *Pseudomonas aeruginosa* stimulation [22]. To study the link between CFTR and NFκB activation, we over-expressed wt-CFTR in HEK293 cells and verified transfection efficiency by cell surface immunofluorescence microscopy. We tested the response to IL1-β, TNFα and *Pseudomonas aeruginosa* LPS by induction of NFκB- or IL-8- reporter activities (1.2–2 fold) and selected IL1-β for further *in vitro* studies based on its prominent and stable proinflammatory stimulus. In baseline conditions and after stimulation with IL-1β, IL-8 and NFκB (Fig 1) promoter-driven luciferase expression was suppressed greater than 2 fold by wt-CFTR as compared to the control cells transfected with empty vector (p<0.05). In cells co-transfected with the IL-8-ΔNFκB promoter, in which we deleted the NFκB transcription site, wt-CFTR no longer reduced the IL-8 promoter activity. We also verified these results by quantitation of basal and IL1-β induced IL-8 chemokine secretion in CFBE41o- and CFBE41o-wt-CFTR cells. Stable wt-CFTR expression blunts the basal (p<0.05) and IL1-β induced IL-8 chemokine secretion in CFBE41o- cells (Fig 2). Our results confirm that CFTR negatively regulates NFκB mediated IL-8 signaling.

### Localization of CFTR in lipid rafts is required to regulate NFκB and IL-8 signaling

**Figure 3:**
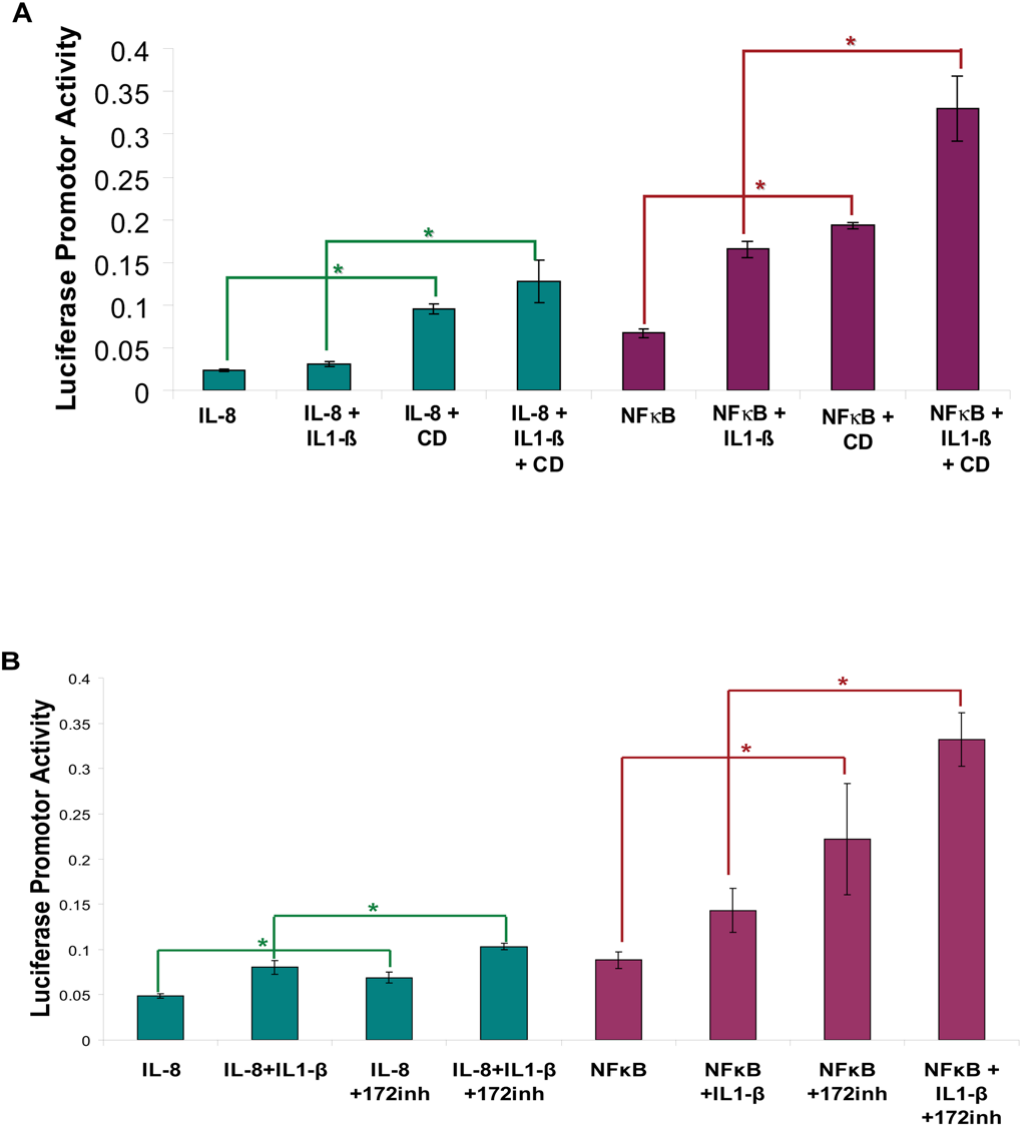
Inhibition of CFTR regulated NFκB signaling under conditions that disrupt lipid rafts. CFBE41o- cells stably transduced with wt-CFTR were transiently transfected with IL-8 or NFκB reporter constructs and a renila luciferase internal control plasmid (n = 3). The cells were induced with 1 ng/ml IL1-β and/or treated with 5 mM methyl-β-cyclodextrin (CD) for 6 hrs (A), and/or 10 µM CFTR-172 inhibitor overnight (B). The data are shown as mean±SD of IL-8 or NFκB promoter activities normalized to renila luciferase internal control. A. IL-1β induced IL-8 and NFκB promoter driven luciferase expression, and wt-CFTR dampened baseline and cytokine induced reporter activity (Figs 1&2). Pre-treatment with CD under conditions known to disrupt lipid rafts eliminated the effect of wt-CFTR, *p<0.05. B. Cells were transfected as in A but instead of treatment with CD, the CFTR-172 inhibitor was included to block CFTR-mediated chloride secretion at the plasma membrane. Pre-treatment with CFTR 172 inhibitor induced IL-8 and NFκB promoter activities and eliminated the inhibitory effect of wt-CFTR, *p<0.05.

It was recently reported that methyl-β-cyclodextrin (CD), a membrane cholesterol scavenger reduces both the basal and stimulated amount of CFTR in detergent resistant membranes (DRMs)[23]. The absence of CFTR in DRMs was associated with abnormal TNFR1 signaling as verified by no recruitment of TNFR1 and c-Src to lipid rafts in CFTR-ΔTRL cells and loss of regulation of gap junctional communication (GJIC) and IL-8 secretion[23]. These results suggest that localization of CFTR in lipid rafts in association with c-Src and TNFR1 provides a responsive signaling complex to regulate GJIC and cytokine signaling. To verify these results, we used NFκB and IL-8 reporter assays to quantify the impact of CFTR inhibition from DRMs in CFBE41o-wt-CFTR cells. Under conditions that deplete CFTR localization to cholesterol-rich lipid rafts, CD treatment significantly induced (>2 fold; p<0.05) NFκB and IL8 reporter activities in CFBE41o-wtCFTR cells as compared to control untreated cells (Fig 3A). These data confirm that CFTR is a negative regulator of NFκB mediated IL-8 signaling and demonstrates the importance of lipid raft localization of CFTR (Fig S1) for its NFκB-regulatory function. We also observed that there is no change in NFκB reporter activity with CD treatment in HEK293 cells in absence of CFTR indicating that CD modulates lipid-raft mediated NFκB activity through CFTR (Fig S2). We next hypothesized that inhibition of wt-CFTR function with CFTR-172 inhibitor in CFBE41o-wtCFTR cells would produce a similar effect on inflammatory signaling as CD. Treatment with CFTR-172 inhibitor induces NFκB (>2 fold) and IL-8 (≥1.33 fold) reporter activities (Fig 3B, p<0.05) supporting the hypothesis that functional CFTR is required for regulating NFκB mediated IL-8 signaling. Our data predicts that the absence of functional CFTR from lipid rafts or cell surface may contribute to a chronic inflammatory state in the presence of *Pseudomonas aeruginosa* infection in human CF subjects or murine model.

### CFTR is required for recovery from LPS induced inflammation

**Figure 4:**
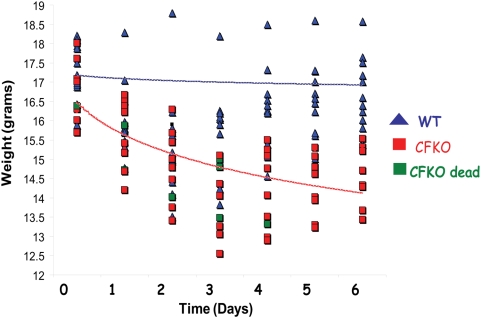
The CFTR knock out mice show significant decline in body weight after LPS treatment. The FABP-CFTR gut corrected CFTR knock-outs (CFKO) and wt- mice (n = 12) were injected i.p. with 15 mg/kg body weight of *P. aeruginosa* LPS, and body weights were recorded daily for 6 days. The CFKO mice show significant decline in body weight as compared to wild type mice (p<0.001; day 3–6). Wild type mice (blue) recover from weight loss by day 6 while CFKO mice (red) don't show significant recovery. The 25% of CFKO mice died during the course of experiment due to LPS induced chronic inflammation (green). The data suggest that CFTR is critical for LPS induced immune response and recovery from LPS induced inflammation. The data are shown as body weight in grams of each mouse in wt- and CFKO- group from day 0 to 6. The non-linear logarithmic regression was used to calculate the trend lines indicating the changes in body weight in wild type (blue) and CFKO (red) mice after LPS exposure.

**Figure 5:**
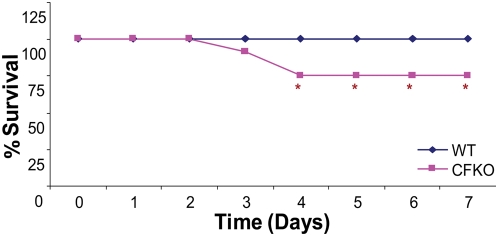
The CFTR knock out mice show significant decline in recovery from inflammation after LPS treatment. The FABP-CFTR gut corrected CFTR knock-outs (CFKO) and wt- mice (n = 12) were injected i.p. with 15 mg/kg body weight of *P. aeruginosa* LPS, and survival was recorded daily for 7 days. The data are shown as percentage of mice that survived at each time point. The CFKO mice (red) show a 25% decline in survival as compared to wild type mice (blue) by day 4 (*p<0.05). Wild type mice show 100% survival and recovery from inflammation at the given LPS dose while 25% of CFKO mice fail to recover and don't survive.

To test the hypothesis that CFTR regulates the proinflammatory response and absence of CFTR results in chronic inflammation in presence of *P. aeruginosa*, we quantified changes in LPS induced systemic immune response in FABP-gut corrected CFTR knock out (CFKO) and wild type (wt-) mice. Mice (n = 12) were injected with *P. aeruginosa* LPS or live bacteria as described in the Methods. We compared body weight, survival and pro-inflammatory response in sera and whole lung protein over 7 days in CFKO mice as compared to wild type controls. Wt- and CFKO- mice both experience a significant (p<0.05) decrease in body weight (Fig 4) although wt- mice can recover from weight loss by day 7 but CFKO mice do not. The box and whisker plot analysis of data was used to determine the variation and central tendency of the replicates (body weights) at each time point (Fig S3). Even more striking is the observation that CFKO mice have 25% lower survival (p<0.05) as compared to their wt- counterparts at similar LPS doses (Fig 5). The inability of CFKO mice to recover from weight loss and decreased survival suggest that CFTR is required for systemic immune response to LPS. Both our *in vitro* (Fig 1, 2&3) and *in vivo* (Fig 4&5) data clearly indicate the negative correlation between CFTR cell surface levels and NFκB mediated inflammatory signaling.

### CFTR negatively regulates IκB-NFκB mediated inflammatory signaling in murine lung

**Figure 6:**
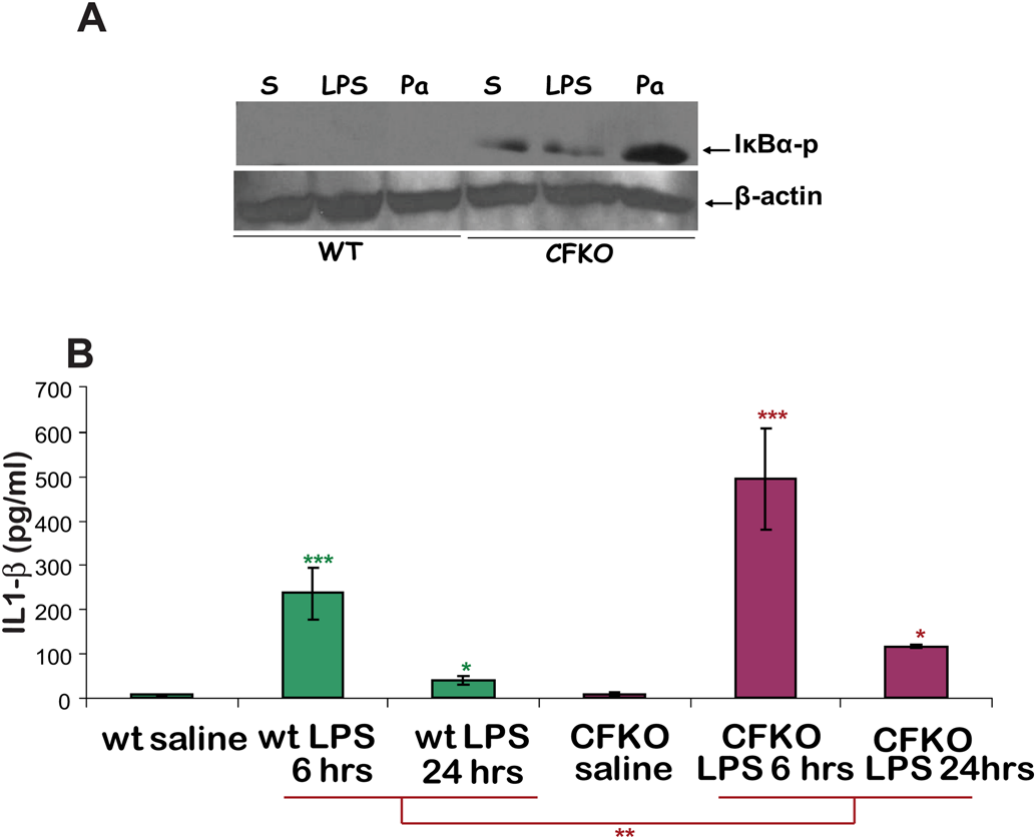
The CFTR knock out mice have elevated IκB-NFκB mediated inflammatory signaling. The FABP-CFTR gut corrected CFTR knock-outs (CFKO) and wt- mice (n = 3) were injected i.p. with 15 mg/kg body weight of *P. aeruginosa* LPS or live bacteria (100 µl, 2×10^8^), and IκB-p levels (24 hrs) were quantified by immunoblotting while serum IL1-β levels (6 or 24 hrs) were determined by ELISA. A. The representative immunoblot indicates the IκB-p protein levels in each group. The lung protein extracts of CFKO mice show inherent increase in IκB-p levels indicative of elevated NFκB signaling and activation. The densitometry analysis of IκB-p protein levels in CFKO lungs as compared to wild type mice show a 4.5 fold increase (n = 3, p<0.05) at baseline. B. The serum concentration of IL1-β (pg/ml) shown as mean±SD. The CFKO mice (red) have significantly higher pro-inflammatory cytokine, IL1-β, levels (***p<0.001 at 6 hrs while *p<0.05 at 24 hrs) on LPS induction as compared to wild type mice (green; **p<0.01).

We hypothesized that the activated phosphorylated form of IκBα (an inducer of NFκB) would be increased in CF mice exposed to *P. aeruginosa* LPS or bacteria, and that wt-mice would have lower levels because CFTR was negatively regulating signaling via TNFα-IL-1β-TLR. We measured phosphorylated IκBα levels in murine lungs by immunoblotting of whole lung protein extracts from the CFKO- and wt- mice. As can be seen in Fig 6A&B, there was a significant increase in phosphorylated IκBα from CFKO lung (p<0.01), as compared to the wild types (n = 3). At this exposure level of the blot we did not see any signal from the wt-mice. We quantified 4.5-fold (p<0.05, n = 3) increase in IκBα-p protein levels in CFKO lungs over wt- that verifies the significant increase in baseline IκBα-p levels in CFKO mice. The proinflammatory stimulus (LPS or bacteria), do not aggravate this increase. We then measured changes in downstream proinflammatory signaling by quantifying IL1-β cytokine levels and observed a steep increase by 6 hrs of LPS treatment. Both 6 and 24 hrs LPS treatments result in significantly higher IL1-β cytokine levels in CFKO mice as compared to the wild type (Fig 6B). We also observed a significant induction of IL1-β by 24 hrs of *P. aeruginosa* LPS in ΔF508- and wt- mice (∼1.2–2 fold; p<0.01) although this induction was much lower than seen in CFKO (∼10 fold; p<0.01) in a parallel experiment. Moreover, ΔF508-mice showed 100% survival after LPS induced inflammation, similar to the wild types. This observation can be explained by a recent report that demonstrates that murine ΔF508-CFTR is more efficient at trafficking to the cell surface when compared to human ΔF508- CFTR [24]. Hence we suspect that ΔF508-mice fail to mimic the severe inflammatory phenotype seen in humans with CF in part because the trafficking of murine ΔF508- is more efficient. [24].

## Discussion

The cystic fibrosis transmembrane conductance regulator (CFTR) gene was identified nearly two decades ago, however it remains unclear whether chloride transport through CFTR is a critical regulator of both epithelial fluid balance and inflammatory signaling. Numerous studies have shown that inflammatory signaling through the NFκB pathway is increased in CF lungs and that this is linked to the production of pro-inflammatory cytokines such as interleukin-8 (IL-8)[25]–[29]. However, there is little consensus on the mechanism(s) which link CFTR or its heritable mutant forms to chronic lung inflammation. In this study, we tested the hypothesis that wild-type CFTR regulates NFκB activity and IL-8 chemokine secretion. Our results show that CFTR cell surface expression, lipid raft localization and uninhibited channel function correlates inversely with NFκB and IL-8 promoter activities, and downstream inflammatory signaling.

We first demonstrated that in both unpolarized and polarized epithelial cells, CFTR is a negative regulator of NFκB mediated inflammatory signaling. In a much more complex *in vivo* system, murine model, we confirmed that CFTR is a negative regulator of the NFκB mediated response and absence of CFTR results in an inherent defect in IκB-NFκB mediated innate immune response. To investigate the precise role of CFTR in regulation of NFκB mediated IL-8 signaling we quantified NFκB and IL-8 reporter activities in HEK293 and CFBE41o- cells in the presence and absence of wt-CFTR. We observed that wt-CFTR negatively regulates both NFκB and IL-8 promoter activities (Fig 1A&B). We also confirmed that CFTR regulates IL-8 reporter activity *via* NFκB by using ΔNFκB-IL-8 promoter and observed that deletion of NFκB site in IL-8 promoter releases the inhibitory effect of CFTR on IL-8 promoter activity. Moreover, we demonstrate that CFTR localization to lipid rafts (Fig S1) is involved in control of inflammation (Fig 3A). Our data supports the view that in cystic fibrosis (CF) patients with ΔF508-mutation, the absence of CFTR from lipid rafts or the cell surface results in chronic inflammation and lung disease. This data indicates that CFTR has anti-inflammatory properties and that the hyper-inflammation found in CF is in part due to a disruption of the signaling link between CFTR and NFκB. This is contrary to the concept that inflammation in the CF lung is entirely due to the loss of chloride and sodium transport causing impaired mucociliary clearance. Further work is required to determine whether rescue of a partially functional CFTR will favorably impact the balance of physiological and inflammatory pathways. A better understanding of these pathways could lead to new treatments for inflammation in CF, resulting in prolonged better quality of life for these patients.

Although it is currently not clear how mutations in CFTR lead to abnormalities of the NFκB pathway, our findings demonstrate that lack of functional CFTR on cell surface and not just accumulation of misfolded CFTR in the endoplasmic reticulum or some other by-product of the CTFR mutation leads to abnormal function of the NFκB pathway. Weber *et al.* recently evaluated cells with CFTR mutations that produce proteins that are trafficked normally to the cell membrane but lack Cl^−^ channel function. The G551D and ΔF508 mutations were associated with up regulation of NFκB activation and increased production of IL-8[30]. They concluded that cell lines with defective CFTR Cl^−^ channel activity, regardless of the type of CFTR defect, have a proinflammatory phenotype. Elucidating the mechanisms by which abnormal Cl^−^ channel function deregulates NFκB activation in CF is an important area for further investigation. Recently, Marc Chanson, Bruce Stanton and colleagues demonstrated that deletion of the PDZ binding domain of CFTR (CFTR-ΔTRL) not only compromises the ability of CFTR to localize to gap junction TNFα protein-complex but also results in activation of downstream NFκB signaling. The absence of CFTR in detergent resistant membranes (DRMs) was associated with abnormal TNFR1 signaling as verified by lack of recruitment of TNFR1 and c-Src to lipid rafts in CFTR-ΔTRL cells and loss of regulation of gap junctional communication (GJIC) and IL-8 secretion[23]. These results suggest that localization of CFTR in detergent resistant and cholesterol rich lipid rafts in association with c-Src and TNFR1 provides a responsive signaling complex to regulate GJIC and NFκB mediated cytokine signaling. This supports the idea that when ΔF508 CFTR is inefficient in trafficking, there is exaggerated NFκB mediated signaling due to lack of gap junctional communication (GJIC) of CFTR with TNFα or IL1-β receptors. We and others have observed that transient or stable over expression of ΔF508-CFTR in HEK-293 or CFBE cells results in decreased NFκB transcriptional activity (Fig S4), but expression of another type of CFTR mutation does not[31]–[33]. In spite of the fact that over expression of misfolded ΔF508 CFTR results in endoplasmic reticulum overload and UPR activation[31]–[33], we anticipate that cell surface expression of over expressed mutant CFTR will inhibit the NFκB activity (as seen in Fig S4) and this warrants further investigation.

**Figure 7:**
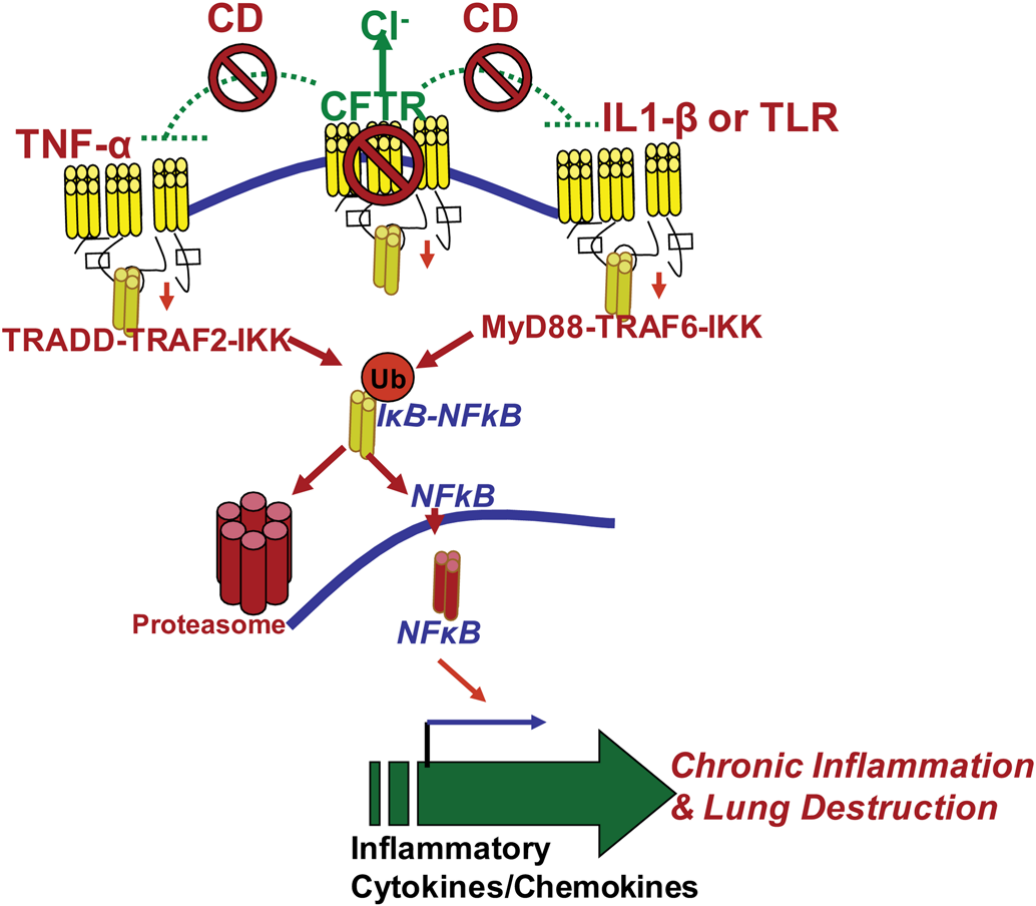
Hypothetical model of CFTR mediated NFκB signaling and innate immune response. The lack of functional CFTR on the cell surface or inhibition of lipid raft localization by methyl-β-cyclodextrin (CD) de-regulates pro-inflammatory signaling *via* TNFα-IL1β-TLR pathways resulting in an overactive innate immune response, chronic NFκB mediated inflammation, and lung destruction. Our data suggest that localization of functional CFTR at the cell surface in cholesterol rich lipid rafts serves as a negative regulator of NFκB signaling. We propose that restoration of an optimal amount of functional CFTR on the cell surface can control chronic inflammatory pathophysiology of CF lung disease by not only restoring the chloride efflux function but by also regulating the chronic NFκB mediated inflammatory signaling.

NFκB mediated IL-8 chemokine secretion and neutrophil influx, are prominent and early features of CF. It is proposed that airway inflammation may occur before or in the absence of bacterial infection. Several recent reports have demonstrated that lung epithelial cells expressing mutant CFTR have increased production of proinflammatory cytokines and exaggerated NFκB-activation[25]–[29]. GB Pier and colleagues have demonstrated a role for CFTR in airway epithelial cell endocytosis of *Pseudomonas aeruginosa*. They proposed that CFTR is a pattern recognition molecule that extracts *P. aeruginosa* LPS from outer membrane into epithelial cells and activates NFκB signaling [34], [35]. They hypothesize that the lack of this initial IL1-β-NFκB proinflammatory signaling[36] in ΔF508- CF patients results in chronic airway inflammation. In contrast, other groups believe that CFTR dysfunction in CF results in exaggerated NFκB signaling that leads to chronic lung disease. Our data for the first time demonstrate that CFKO mice have inherently higher levels of phosphorylated IκB (Fig 6A) supporting the notion that lack of CFTR results in hyper-inflammatory signaling by compromising the regulatory mechanisms of innate immunity. In addition, we observed that cell surface localization of CFTR is required for regulation of gap junction communication (GJIC) and response to pathogen associated molecular patterns (PAMPs) by primary signaling receptors (TNFα, IL1-β or TLRs) of innate defense (Fig 3A&6 and Fig S1). This may explain why mutations or single nucleotide polymorphisms (SNPs) of these receptors have the modifier function in CF or CF like diseases[37]–[39]. Our *in vitro* and *in vivo* data clearly support the hypothesis that CFTR serves as a negative regulator of innate immunity and both CFTR channel function and its localization to lipid rafts are critical for controlling NFκB mediated inflammatory signaling (Fig 7).

Recent studies have shown that localization of CFTR to DRMs increases in presence of *P. aeruginosa* or proinflammatory stimulus[23], [40], we anticipate that this may be a homeostatic mechanism to control inflammation. We also observed that not only CFTR channel expression and its localization to DRMs but also its channel function is required to control inflammation. There can be two possible scenarios where CFTR channel function can regulate proinflammatory response: 1.) cell surface or 2.) DRMs. The mechanism of first scenario is apparent but it is not clear if functional CFTR is required in DRMs, we predict that non-functional channels may be incapable of localization to DRMs in presence of proinflammatory stimulus resulting in uncontrolled inflammatory response. We are further investigating the role of CFTR channel function in its DRM localization.

Together, these studies suggest a mechanism that links CFTR localization in lipid rafts, and channel function to intracellular NFκB signaling and inflammatory response. The clinical implication of these findings is that treatment of CF patients with small molecule or therapeutic compounds that rescues optimal amount of mutant CFTR to the cholestrol-rich cell surface lipid rafts can inhibit the NFκB mediated chronic inflammation and rescue the pathology induced by defective CFTR, potentially attenuating the progression of CF or related obstructive lung diseases like COPD and emphysema.

## Materials and Methods

### Cell Culture and Reagents

The HEK293, CFBE41o- (cystic fibrosis bronchial epithelial cell lines, originally immortalized and characterized by Dr. Dieter Gruenert[41], [42]), CFBE41o-wtCFTR or CFBE41o-ΔF08-CFTR (stably transduced with wt-CFTR or ΔF08-CFTR by Dr. JP Clancy[43]) cell lines were maintained in DMEM/F12 and MEM Earl's salt L-Glutamine (200 mM L-Glutamine) medium containing 100 units/ml penicillin, 100 µg/ml streptomycin, 0.25 µg/ml amphotericin B and 10% fetal bovine serum. The CFBE41o-wtCFTR or CFBE41o-ΔF08-CFTR cells were always cultured in presence of 500 µg/ml Hygromycin B. DMEM/F12, MEM and other components were purchased from Gibco or Invitrogen, Carlsbad, CA. TNF-α, IL-1β (R&D Systems Inc., Minneapolis, MN), CFTR-172 inhibitor (Calbiochem, Gibbstown, NJ), methyl-β-cyclodextrin and *Pseudomonas aeruginosa* LPS (Sigma, St. Louis, MO) were added to cells or injected in mice as indicated. *Pseudomonas aeruginosa* PAO1 strain was obtained from ATCC for animal experiments. Sulfo-NHS-SS-Biotin was purchased from Pierce, Rockford, IL while other common laboratory chemicals were from Sigma.

### Immunoblotting

Lung tissues were lysed by sonication (three 5 sec pulses) on ice in cold room using the T-PER (Pierce Biotech. Inc., Rockford, IL) protein lysis buffer containing protease-inhibitor cocktail (Pierce). The protein extracts were suspended in Laemmli's sample buffer (Invitrogen) containing β-mercaptoethanol (Invitrogen), resolved by 4–10% SDS-PAGE and transferred to a 0.45 µm pore size nitrocellulose membrane (Invitrogen). The β-actin (Sigma) and IκB-p (Cell Signaling, Danvers, MA) primary antibodies, and anti-rabbit-HRP secondary antibody (Amersham, Piscataway, NJ) were used for immunoblotting.

### Animal Experiments

All animal experiments were carried out in accordance with the Johns Hopkins University (JHU) Animal Care and Use Committee (ACUC) approved protocol. To measure inflammatory signaling *in vivo*, the age and sex matched C57BL6, FABP-CFTR gut corrected CFTR knock-outs, ΔF508-homozygous and wt- mice (n = 12) were injected i.p. with 15 mg/kg body weight of *P. aeruginosa* LPS, and survival and body weights were recorded daily for 6–7 days. Serum and total lung protein extracts were isolated at 6 and 24 hrs time points in a parallel experiment. Serum IL1-β cytokine levels were quantified by sandwich ELISA (described below) to identify the changes in pro-inflammatory signaling. The total lung protein extracts from both *P. aeruginosa* LPS and live bacteria (2×10^8^) injected (i.p.) mice were used for quantification of IκB-p and β-actin protein levels by Western blotting.

### IL-1β and IL-8 Immunoassay

At the indicated time points, serum was collected and IL-1β levels were measured using solid-phase ELISA (R&D Bisoystems, Minneapolis, MN). Standards, and high and low cytokine controls were included. The plates were read at 450 nm on 96-well microplate reader (Molecular Devices, Sunnyvale, CA) using SOFT-MAX-Pro software (Molecular Devices). The mean blank reading was subtracted from each sample and control reading. The amount of substrate turnover was determined calorimetrically by measuring the absorbance which is proportional to IL-1β concentration. A standard curve was plotted and an IL-1β concentration in each sample was determined by interpolation from standard curve. The data represents the mean of three independent experiments±SD. The IL-8 chemokine secretion in media was similarly quantified using an EASIA system (Invitrogen) as described before[18].

### Transfection, NFκB- and IL-8- Reporter Assays

CFBE41o- or HEK293 cells were transfected with NFκB- or IL-8- (with or without the NFκB site) firefly luciferase promoter (pGL-2) and renila luciferase (pRLTK) control, or CFTR (wt- or ΔF508- GFP) constructs. Renila luciferase internal control was used for normalization of DNA and transfection efficiency of reporter constructs in all experiments. Transfection efficiency of CFTR constructs and surface localization of wt-CFTR was verified by immunofluorescence microscopy. Cells were induced with 1 ng/ml of IL1-β, TNF-α or *Pseudomonas aeruginosa* LPS and luciferase activities were measured after overnight treatment. The 10 µM CFTR-172 inhibitor overnight or 5 mM methyl-β-cyclodextrin (CD) 6 hrs, treatment was used to inhibit the wt-CFTR function or its localization to lipid rafts, respectively. Dual-Luciferase® Reporter (DLRTM) Assay System (Promega) was used to measure NFκB or IL-8 reporter (firefly luciferase) and renila luciferace activities from HEK293 or CFBE41o- cell extracts. Data was normalized with internal renila luciferase control for each sample and the changes in reporter activities with CFTR over expression were calculated.

### Statistical Analysis

Representative data is shown as the mean±SD of at least three experiments. The one-way ANOVA with a Dunnett planned comparison was run for each sample versus control. The Box and Whisker plot analysis was used to determine the variation in body weights overtime and central inclination of data among the two genotypes. A *p<0.05 was considered to have statistical significance.

## Supporting Information

Figure S1 In the absence of CFTR, disruption of lipid rafts does not modulate NFκB signaling. HEK293 cells were transiently transfected with NFκB reporter construct and a renila luciferase internal control plasmid (n = 3). The cells were induced with 1 ng/ml IL1-β and/or treated with 5 mM methyl-β-cyclodextrin (CD) for 6 hrs. The data are shown as mean±SD of NFκB promoter activity normalized to renila luciferase internal control. IL-1β induced NFκB promoter driven luciferase expression, and CD treatment under conditions known to disrupt lipid rafts has no significant effect on NFκB promoter activity in the absence of CFTR.
(0.69 MB EPS)

Figure S2 Box and whisker plot analysis of the CFTR knock out mice show significant decline in body weight after LPS treatment. The Box and whisker plot analysis was used to determine the variation and central tendency of data at each time point. The box represents the distance between the 1st and 3rd quartiles (Q). The whiskers show the highest and lowest data points or 1.5 times the box (Q3-Q1). Outlier points (X) are those that are greater than 1.5 times (Q3 -Q1). The yellow box represents the difference between the Q3 and the median while green box indicates the difference between the Q1 and the median. The data shows the statistical significance of the decline in body weights of FABP-CFTR gut corrected CFTR knock-outs (CFKO) mice as compared to the wild type mice (n = 12) from day 0 to 6. (A) Wild type mice recover from weight loss by day 6 while (B) CFKO mice don't show significant recovery.
(1.19 MB EPS)

Figure S3 Disruption of detergent resistant membranes (DRMs) by methyl-β-cyclodextrin (CD) inhibits raft CFTR. CFBE41o- cells stably transduced with wt-CFTR were treated with 5 mM methyl-β-cyclodextrin (CD) for 6 hrs. The plasma membrane proteins biotinylated with 1 mg/ml sulfo-NHS-SS-biotin for 60 min at 37°C were isolated by Streptavidin-Sepharose pull down. The sucrose density gradient of these biotin labeled membrane proteins was used to isolate lipid raft and soluble fractions followed by CFTR immunoblotting. CD treatment significantly reduces the amount of raft CFTR but has no effect on soluble CFTR. The densitometric analysis (lower panel) of the raft CFTR bands illustrates the inhibitory effect of CD on CFTR localization to lipid raft. The graph shows mean+SD of duplicate experiments (*p<0.05).
(1.26 MB EPS)

Figure S4 ΔF508-CFTR downregulates NFκB mediated IL-8 reporter activity. CFBE41o- controls and cells stably transduced with ΔF508-CFTR were transiently transfected with IL-8 or IL8-ΔNFκB reporter constructs and a renila luciferase internal control plasmid (n = 3). The cells were induced with 1 ng/ml IL1-β overnight. Data are normalized to the internal control and expressed as mean+SD (*p<0.05). IL-1β induced IL-8 promoter expression and ΔF508-CFTR dampened baseline and cytokine induced reporter activity. The deletion of NFκB transcription site from IL-8 promoter (IL8-ΔNFκB) abolished both IL1-β meditated IL-8 induction and inhibitory effect of ΔF508-CFTR.
(0.75 MB EPS)
